# Dual mediating effects of learning motivation and hope on the relationship between generative AI acceptance and learning engagement among Chinese college students: moderated mediation effect of self-directed learning

**DOI:** 10.3389/fpsyg.2026.1842819

**Published:** 2026-07-21

**Authors:** Yinshun Zhang, Chang Seek Lee, Chao Deng, Xin Liao

**Affiliations:** 1 School of Financial Technology and Accounting, Guangdong University of Science and Technology, Dongguan, Guangdong, China; 2 Department of Convergence Lifelong Education, Hanseo University, Seosan, Republic of Korea; 3 Department of Lifelong Education, Hanseo University, Seosan, Republic of Korea; 4 School of Computer Science and Artificial Intelligence (Industrial Software), Guangdong University of Science and Technology, Dongguan, Guangdong, China; 5 Department of Computer Science and Technology, Guangdong University of Technology, Guangzhou, China

**Keywords:** college student, generative AI acceptance, hope, learning engagement, learning motivation, moderated mediation effect, self-directed learning

## Abstract

In recent years, generative artificial intelligence (GenAI) has attracted growing attention in higher education; however, the psychological mechanisms through which generative AI acceptance influences students’ learning engagement remain insufficiently understood. This study aimed to examine a moderated sequential mediation model linking generative AI acceptance, learning motivation, hope, learning engagement, and self-directed learning. To this end, the study developed and empirically tested a moderated mediation model via a survey-based research design. Following informed consent procedures, data from 478 Chinese university students were collected using an online questionnaire platform. Data were analyzed using SPSS (v26.0), PROCESS macro (v4.2), and AMOS (v24.0), encompassing descriptive statistics, reliability and validity assessments, confirmatory factor analysis, correlation analysis, and moderated mediation analysis. Results revealed that generative AI acceptance, learning motivation, hope, self-directed learning, and learning engagement were all positively correlated. Moreover, learning motivation and hope served as sequential mediators in the relationship between generative AI acceptance and learning engagement. Furthermore, self-directed learning significantly moderated the path from learning motivation to hope, and the overall moderated mediation effect was statistically significant. These findings provide novel insights into the association between generative AI acceptance and learning engagement by highlighting the roles of learning motivation, hope, and self-directed learning. Specifically, the findings support a theoretically derived sequential pathway in which learning motivation and hope are associated with the relationship between generative AI acceptance and learning engagement, while self-directed learning moderates this pathway. This study contributes to the technology-enhanced learning literature by extending understanding of these relationships and offers practical implications for supporting effective AI-assisted learning in higher education.

## 1 Introduction

Learning engagement, broadly defined as a psychological state reflecting students’ active involvement in the learning process, encompasses behavioral, cognitive, and emotional dimensions (Fredricks et al., 2004). Rather than merely focusing on observable academic actions, this construct encompasses active psychological processes, proactive attitudes, and deep cognitive reflection. Theoretically, learning engagement not only drives academic achievement and deep learning but also promotes students’ psychological well-being and holistic development (Fredricks et al., 2004). Consistently, Hattie (2003) argued that effective learning requires such active engagement and continuous reflection, rather than a reliance on rote memorization.

The rapid advancement of generative artificial intelligence, exemplified by platforms such as ChatGPT and DeepSeek, has profoundly reshaped higher education by offering unprecedented opportunities to personalize learning, enrich feedback, and support students’ cognitive and emotional development (Kasneci et al., 2023). As these technologies become increasingly embedded in academic environments, a critical need emerges to understand the processes through which students’ acceptance of generative AI may be associated with meaningful learning outcomes, particularly learning engagement (An et al., 2022). Grounded in the Technology Acceptance Model (TAM), prior research has demonstrated that perceived usefulness and ease of use predict learners’ behavioral intentions toward educational technologies (Davis, 1989; Venkatesh et al., 2003). However, growing evidence suggests that the relationship between technology acceptance and learning outcomes is rarely direct; rather, it is mediated by learners’ internal psychological processes (Scherer et al., 2019). Despite the rapid diffusion of GenAI in education, the specific motivational and emotional mechanisms linking generative AI acceptance to learning engagement remain insufficiently understood.

Learning motivation serves as a pivotal psychological mechanism that drives academic behaviors and goal-directed pursuits. Grounded in self-determination theory, educational technologies perceived as autonomy-supportive and competence-enhancing can foster intrinsic motivation by fulfilling learners’ basic psychological needs (Ryan and Deci, 2020). Consistently, accumulating empirical evidence indicates that digital tool acceptance positively predicts learning motivation, which in turn facilitates learning engagement (Yang, 2025). Within digital and online learning environments, robust motivation has been shown to directly determine both cognitive engagement and metacognitive regulation strategies (Hua, 2019). Furthermore, recent empirical insights confirm that technology acceptance indirectly enhances learning engagement by boosting students’ intrinsic motivation (An et al., 2024). Consequently, learning motivation acts as a critical psychological bridge connecting initial technology acceptance to sustained behavioral and cognitive investment.

Nevertheless, motivation alone may not fully account for how students maintain sustained engagement, particularly within generative AI-supported environments that demand continuous self-regulation and cognitive adaptation. To address this gap, hope conceptualized as a future-oriented positive psychological resource, emerges as a critical affective determinant. Hope theory posits that high-hope individuals possess both agency (goal-directed determination) and pathways (planning to meet goals), enabling them to persist despite academic obstacles (Snyder, 2002). Prior research indicates that learning motivation fosters hope by clarifying goals and enhancing perceived goal attainability, which subsequently facilitates heightened learning engagement (Hertinjung et al., 2025; Snyder, 2002). However, empirical evidence regarding hope as an underlying mechanism linking motivation to engagement remains limited in technology-mediated or GenAI-supported contexts.

Moreover, individual differences in self-directed learning (SDL) likely moderate how these motivational resources translate into positive emotional experiences. SDL refers to learners’ capacity to independently establish goals, monitor progress, and adjust learning strategies (Garrison, 1997). In GenAI-supported settings, students with high SDL are better positioned to leverage AI tools strategically, thereby translating intrinsic motivation into hopeful expectations of goal attainment. While empirical evidence confirms that SDL strengthens the link between motivational beliefs and academic outcomes, serving as a critical boundary condition (Broadbent and Poon, 2015), its specific moderating role within the motivational and affective pathways underlying GenAI adoption warrants further investigation.

To address these critical gaps, the present study proposes a moderated chain mediation model to examine the complex interrelationships among generative AI acceptance, learning motivation, hope, learning engagement, and self-directed learning. Specifically, this study investigates whether learning motivation and hope sequentially mediate the effect of generative AI acceptance on learning engagement. Furthermore, it evaluates the boundary condition of self-directed learning by testing its potential to moderate the psychological pathway from learning motivation to hope.

## 2 Literature review

### 2.1 The relationship between generative AI acceptance and learning engagement

With the increasing prevalence of generative artificial intelligence in higher education, generative AI acceptance has become a key variable for predicting students’ technology adoption behavior. According to the Technology Acceptance Model (TAM), learners’ perceived ease of use and perceived usefulness of new technologies shape their behavioral intention to adopt them (Davis, 1989). In this context, generative AI acceptance extends beyond mere tool adoption; it also reflects students’ willingness to integrate AI into their cognitive processes (Kalmus and Nikiforova, 2024; Shrivastava, 2025).

Learning engagement encompasses the behavioral, emotional, and cognitive dimensions of students’ involvement in learning activities (Fredricks et al., 2004). Existing research shows that when students accept and actively adopt GenAI tools such as ChatGPT and Midjourney, they tend to report greater learning self-efficacy and consequently invest more time and effort in deep learning (Chiu, 2024). Drawing on the Unified Theory of Acceptance and Use of Technology (UTAUT) and learning engagement theory, Pan and He (2024) found that technology acceptance positively predicted learning engagement. However, the direct relationship between generative AI acceptance and learning engagement remains empirically contested. This can be understood through the Productivity Paradox (Brynjolfsson, 1993), which posits that the adoption of advanced technologies does not automatically lead to improved performance outcomes. Rather, the benefits of technological resources are realized through complementary psychological, organizational, and human factors (Brynjolfsson and Hitt, 1996). In educational settings, students’ acceptance of generative AI may create learning opportunities, but enhanced learning engagement is unlikely to occur unless learners develop sufficient motivation and positive psychological resources, including hope. As Lai and Bower (2019) argued, technology acceptance provides only the possibility of engagement, while meaningful engagement depends on how learners apply technology through specific learning strategies. Luo et al. (2025) found that although AI technology can provide immediate feedback, students’ engagement declines when they perceive it as a tool for academic dishonesty rather than a learning aid. Therefore, investigating the underlying mechanisms through which generative AI acceptance influences learning engagement—notably motivational processes and positive psychological resources—is of considerable theoretical and practical importance.

### 2.2 The dual mediating roles of learning motivation and hope

Learning motivation is a foundational construct in educational psychology, reflecting the internal and external forces that initiate, direct, and maintain learning behaviors (Ryan and Deci, 2020). In digital learning contexts, students’ acceptance of technology has been shown to enhance learning motivation by fostering perceived autonomy, competence, and task relevance (Hartnett et al., 2023). Generative AI tools, by offering immediate feedback and personalized assistance, may further strengthen students’ motivation by facilitating goal attainment and alleviating learning frustration (Cengiz and Peker, 2025; Shrivastava, 2025). Wang and Eccles (2012) found that students with high levels of intrinsic motivation exhibited greater attention, involvement, and depth of cognitive processing during classroom activities. Moreover, interest in learning—a core component of intrinsic motivation—has been shown associated with students’ proactive inquiry and persistence in complex learning tasks (Hidi and Renninger, 2006).

Motivated learners are more likely to experience positive emotional states that support sustained learning engagement. Among these emotional factors, hope has emerged as a particularly important future-oriented psychological resource. According to hope theory, hope consists of agency thinking (the motivation to pursue goals) and pathways thinking (the perceived ability to generate routes toward those goals) (Snyder, 2002). Learning motivation can foster hope by strengthening students’ confidence in their capacity to achieve learning goals and by clarifying viable pathways to goal attainment. Unlike traditional learning resources, which often provide fixed content and delayed feedback, generative AI systems can offer immediate, personalized, and adaptive guidance. For example, when students encounter learning difficulties, AI tools can instantly generate alternative explanations, recommend different problem-solving strategies, and provide step-by-step learning pathways. Such functions directly enhance pathways thinking by helping learners identify multiple routes to academic goal attainment. Empirical research has demonstrated that hope positively predicts academic engagement, persistence, and achievement (Gallagher, 2023; Snyder, 2002). Students with higher levels of hope tend to display greater resilience and persistent effort when encountering academic challenges. Recent studies in technology-enhanced learning contexts suggest that motivational beliefs may indirectly influence engagement through positive emotions, although such mechanisms have received limited empirical attention in generative AI settings.

It follows that learning motivation and hope may function as sequential mediators linking generative AI acceptance and learning engagement. Specifically, students with higher levels of generative AI acceptance may also report higher learning motivation, which may, in turn, be associated with greater hope characterized by stronger agency and pathways thinking. Higher levels of hope may further be associated with greater cognitive and behavioral investment in learning activities. Examining this dual mediating mechanism may provide a more comprehensive understanding of the motivational and emotional processes underlying the observed association between generative AI acceptance and learning engagement in AI-supported learning environments.

### 2.3 The moderating role of self-directed learning

Self-directed learning refers to learners’ ability to diagnose learning needs, set goals, select strategies, and evaluate learning outcomes independently (Garrison, 1997). In technology-rich environments, self-directed learning has been identified as a critical factor influencing learners’ ability to effectively use digital tools (Broadbent and Poon, 2015). Growing scholarly attention has been directed toward the relationship between self-directed learning and learning engagement. A mixed-methods study conducted in Chinese universities revealed a significant positive association between students’ self-directed learning capacity and their academic performance and engagement, suggesting that greater self-directed learning ability is associated with higher levels of learning engagement (Li, 2023). Another study confirmed that core elements of self-directed learning, such as academic resilience, curiosity, and self-efficacy, can lead to higher levels of engagement and course completion (George et al., 2022). Sun and Rueda (2012) found that students with stronger self-directed learning abilities demonstrated higher levels of engagement in online learning environments, particularly across cognitive and affective dimensions. Zimmerman (2002) further demonstrated that self-directed learners are more likely to employ metacognitive strategies—such as planning, monitoring, and evaluation—which facilitate deep learning and enhance the quality of cognitive engagement.

Recent empirical evidence suggests that self-directed learning moderates the relationship between motivational beliefs and learning outcomes in digital environments (Liu et al., 2025). This suggests that the effect of learning motivation on hope may be stronger among students with higher levels of self-directed learning. In contrast, students with lower self-directed learning may experience difficulties in maintaining hope despite being motivated, due to limited self-regulatory capacities.

Accordingly, self-directed learning is expected to function as a boundary condition within the motivational and affective pathway linking generative AI acceptance to learning engagement. By moderating the pathway from learning motivation to hope, self-directed learning may determine the strength of the indirect effects of generative AI acceptance on learning engagement. The present study thus aims to develop a theoretically grounded model that elucidates how self-directed learning moderates the motivational and affective pathways underpinning learning engagement in technology-enhanced contexts, thereby addressing critical gaps in the existing literature.

### 2.4 Hypothesis development

Drawing on the foregoing literature review, the following hypotheses are proposed:

*H1*: Generative AI acceptance, learning motivation, hope, and learning engagement are all positively correlated with one another.

*H2*: Learning motivation positively mediates the relationship between generative AI acceptance and learning engagement.

*H3*: Hope positively mediates the relationship between generative AI acceptance and learning engagement.

*H4*: Learning motivation and hope sequentially and positively mediate the relationship between generative AI acceptance and learning engagement.

*H5*: Self-directed learning moderates the relationship between learning motivation and hope.

*H6*: Self-directed learning moderates the indirect effect of generative AI acceptance on learning engagement via the sequential mediation of learning motivation and hope.

## 3 Research methods

### 3.1 Research model

**Figure 1:**
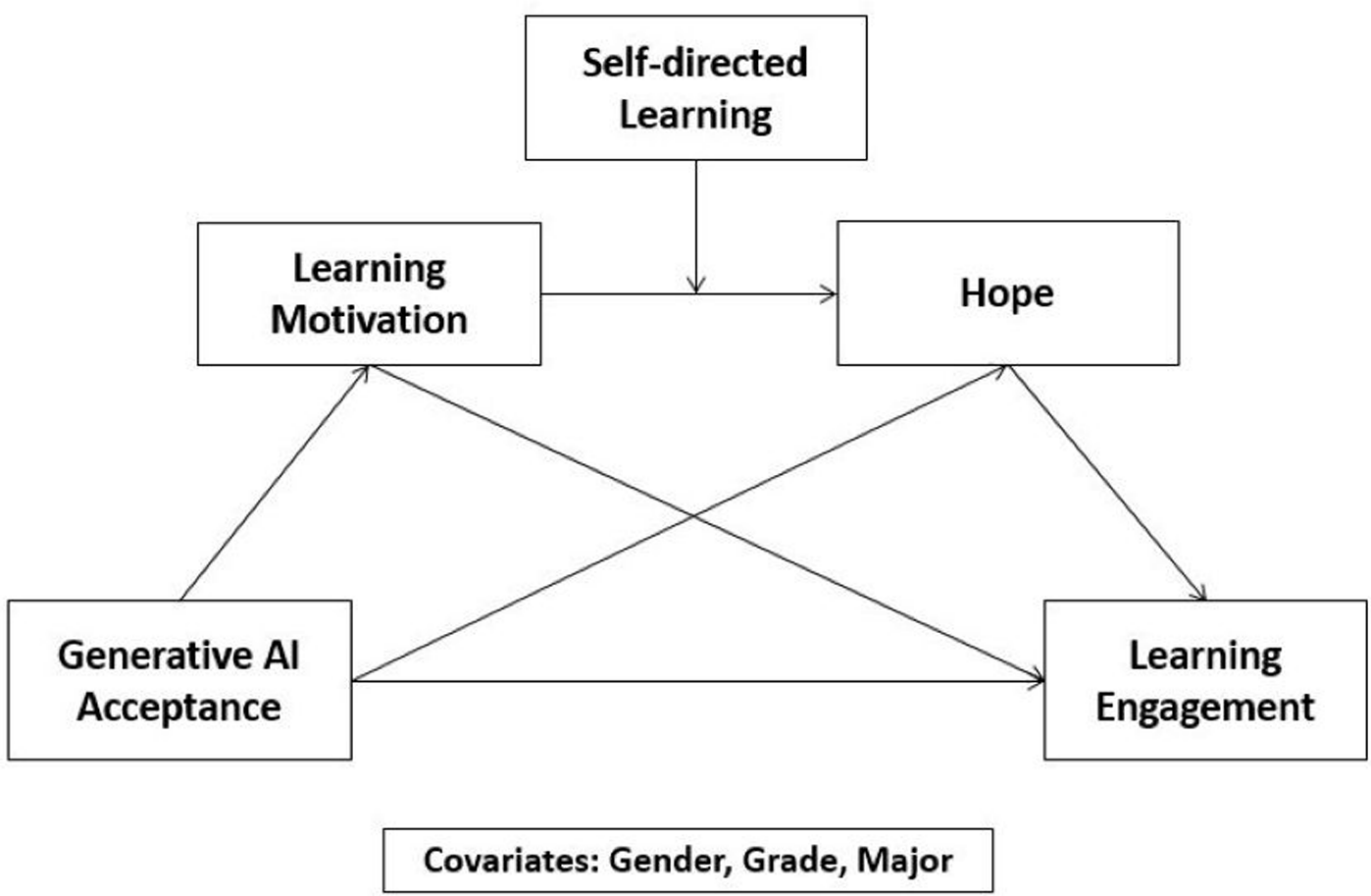
Research model.

The research model (Figure 1) proposed in this study was used to verify the dual mediating role of learning motivation and hope in the path from generative AI acceptance to learning engagement, and then, to prove the moderated mediating role of self-directed learning in the above relationship. Model Number 91 of the SPSS PROCESS macro proposed by Hayes (2017) was applied. Previous research has shown that gender and grade may be related to college students’ learning engagement. To avoid the influence of control variables such as gender, grade, and major, these factors were controlled in subsequent analyses.

### 3.2 Participants and procedures

This study employed a convenience sampling procedure, with the sample covering 32 of the 34 provincial-level administrative regions in China. First, 2,853 registered university students were randomly selected from the Wenjuanxing survey platform database. Each selected student was then sent an invitation package comprising an informed consent form and the study questionnaire. The invitation package outlined the study’s purpose, procedures, potential benefits and risks, confidentiality provisions, voluntary participation, and the right to withdraw at any time without penalty. Only students who provided informed consent were eligible to participate in the study. Of the 2,853 students contacted, 1,216 agreed to participate and provided informed consent. Consenting participants were asked to complete the online questionnaire via the Wenjuanxing platform; of these, 622 submitted their responses within the allotted 20 min time frame. Participants who completed and submitted the questionnaire received a coffee voucher as compensation. Following data screening, incomplete and invalid responses were excluded, yielding a final sample of 478 valid questionnaires.

Of the 478 participants, 199 were male (41.6%), and 279 were female (58.4%). In terms of academic year, 28.7% were first-year students, 37.0% were second-year students, 25.7% were third-year students, and 8.6% were fourth-year students. Regarding academic discipline, 70.7% of participants were enrolled in humanities and social sciences programs, and 29.3% in natural sciences programs. With respect to generative AI usage frequency, 4.8% of participants reported never using it, 25.3% rarely, 47.3% sometimes, 18.8% often, and 3.8% always.

### 3.3 Measuring instruments

#### 3.3.1 Generative AI acceptance

Generative AI acceptance (GAA) was measured using a revised version of the Technology Acceptance Model (TAM)-based scale developed by Zhang (2023) and originally grounded in Davis’s (1989) framework. The original instrument consisted of 17 items (e.g., “Generative AI tools help me save time when searching for information”) assessing students’ acceptance of generative AI technologies. All items were rated on a five-point Likert scale ranging from 1 (strongly disagree) to 5 (strongly agree), with higher scores indicating higher levels of generative AI acceptance. To establish construct validity within the current sample, confirmatory factor analysis (CFA) was conducted. Five items with low factor loadings or substantial cross-loadings were removed during model refinement. The final 12-item measurement model demonstrated a satisfactory fit to the data [*χ*^2^/df = 3.808, CFI = 0.964, TLI = 0.952, RMSEA = 0.077, SRMR = 0.058]. Importantly, although five items were removed, all core conceptual dimensions of the original instrument remained represented in the refined scale, including perceived usefulness, perceived ease of use, perceived risk, social influence, and overall attitudes toward generative AI. Therefore, the content validity of the construct was considered to be preserved.

Reliability analysis based on the refined 12-item scale yielded a Cronbach’s *α* coefficient of 0.906, indicating excellent internal consistency. Convergent validity was assessed at the first-order factor level. The composite reliability (CR) values ranged from 0.867 to 0.909, while the average variance extracted (AVE) values ranged from 0.619 to 0.769, all exceeding the recommended thresholds these indices exceeded the recommended thresholds (e.g., CFI and TLI > 0.90; RMSEA < 0.08; CR > 0.7; AVE > 0.5), confirming that the refined measurement model represents a robust fit for the empirical data.

#### 3.3.2 Learning motivation

The learning motivation (LM) scale in this study used the scale compiled by Chi and Xing (2006). This scale is designed to measure college students’ learning motivation, with items such as “I am willing to try to solve complex learning problems” and “For me, success means doing better than others,” and consists of a total of 16 questions. The measurement was conducted using a 5-point Likert scale, with higher scores indicating higher levels of learning motivation. In this study, the Cronbach’s *α* was 0.937, and the fitting of the validation factor analysis was adequate [*χ*^2^/df = 3.929, CFI = 0.955, TLI = 0.941, RMSEA = 0.078, SRMR = 0.054]. For the multidimensional construct of learning motivation, convergent validity was assessed at the first-order factor level. The composite reliability (CR) values ranged from 0.896 to 0.940, and the average variance extracted (AVE) values ranged from 0.520 to 0.661, confirming that the measurement model represents a robust fit for the empirical data.

#### 3.3.3 Self-directed learning

Self-directed learning (SDL) was measured using the scale developed by Chang (2024). The instrument assesses university students’ self-directed learning abilities and includes items such as “I study according to my own learning plan” and “I have confidence in my learning ability.” The original scale consisted of 14 items covering four dimensions: self-direction, learning strategy application, self-monitoring, and self-evaluation. All items were rated on a five-point Likert scale ranging from 1 (strongly disagree) to 5 (strongly agree), with higher scores indicating higher levels of self-directed learning.

To evaluate the construct validity of the scale in the current sample, confirmatory factor analysis (CFA) was conducted. Two items with substantial cross-loadings were iteratively removed during model refinement to improve model fit and discriminant validity. Importantly, although two items were removed, all four theoretical dimensions remained adequately represented by multiple indicators. Therefore, the conceptual coverage of the self-directed learning construct was preserved, supporting the content validity of the revised scale.

The refined 12-item measurement model demonstrated a satisfactory fit to the data [*χ*^2^/d*f* = 3.675, CFI = 0.982, TLI = 0.970, RMSEA = 0.075, SRMR = 0.031, AVE = 0.660, CR = 0.959]. Reliability analysis based on the final 12-item scale yielded a Cronbach’s *α* coefficient of 0.961, indicating excellent internal consistency. Convergent validity was also supported, with a composite reliability (CR) value of 0.959 and an average variance extracted (AVE) value of 0.660, both exceeding the recommended thresholds of 0.70 and 0.50, respectively (Hair et al., 2022). These results indicate that the revised SDL scale possesses satisfactory reliability and construct validity in the present study.

#### 3.3.4 Hope

Originating from the scale designed by Snyder (2002), this scale consists of 8 items in total, with items such as “I can think of many ways to get out of a jam.” It’s a 5-point Likert scale ranging from “Strongly disagree” to “Strongly agree.” An increasing score represents stronger hope. In this study, the Cronbach’s *α* was 0.931, and the fitting of the validation factor analysis was adequate [*χ*^2^/d*f* = 3.115, CFI = 0.988, TLI = 0.980, RMSEA = 0.067, SRMR = 0.020, AVE = 0.627, CR = 0.930].

#### 3.3.5 Learning engagement

Learning engagement (LE) was measured using the scale developed by Lam et al. (2014). The instrument assesses students’ engagement in learning activities across behavioral, emotional, and cognitive dimensions and includes items such as “At school, I strive to perform well” and “When studying, I decide which key points to focus on rather than reading broadly.” The original scale consisted of 15 items. All items were rated on a five-point Likert scale ranging from 1 (strongly disagree) to 5 (strongly agree), with higher scores indicating higher levels of learning engagement.

To evaluate construct validity within the current sample, confirmatory factor analysis (CFA) was conducted. Four items with substantial cross-loadings or negative factor loadings were iteratively removed during model refinement to improve model fit and discriminant validity. Although several classroom-focused behavioral items and one reverse-worded emotional item were removed, the retained indicators continued to represent the three core dimensions of learning engagement—behavioral engagement, emotional engagement, and cognitive engagement. Furthermore, the remaining items adequately captured students’ persistence, participation, emotional attachment, and cognitive investment in learning activities. Therefore, the conceptual coverage of the construct was preserved, supporting the content validity of the revised instrument.

The refined 11-item measurement model demonstrated a satisfactory fit to the data [*χ*^2^/d*f* = 3.931, CFI = 0.978, TLI = 0.967, RMSEA = 0.078, SRMR = 0.027, AVE = 0.653, CR = 0.953]. Reliability analysis based on the final 11-item scale yielded a Cronbach’s *α* coefficient of 0.954, indicating excellent internal consistency. Convergent validity was also supported, with a composite reliability (CR) value of 0.953 and an average variance extracted (AVE) value of 0.653, both exceeding the recommended thresholds of 0.70 and 0.50, respectively (Hair et al., 2022). Collectively, these findings indicate that the revised LE scale demonstrated satisfactory reliability and construct validity in the present study.

Following established psychometric practices, items with low factor loadings, substantial cross-loadings, or negative loadings were removed during CFA model refinement. Importantly, all retained scales continued to represent the theoretical dimensions of the original instruments, thereby preserving content validity while improving construct validity and model fit.

### 3.4 Data analysis

This study used SPSS 26.0 for data analysis, including descriptive statistics and correlation analysis of variables. Additionally, the Unmeasured Latent Method Construct test was applied to check for common method bias. AMOS 24.0 was used to test the model fit of variables and the research model. Finally, the SPSS PROCESS macro model No. 91 was undertaken to show the moderated dual mediation path among the variables. In addition, to analyze the moderated mediation effect, prior to the analysis, continuous variables were mean-centered, the confidence level of the output confidence interval was 95%, and the number of bootstrap samples for the percentile bootstrap confidence interval was 5,000.

### 3.5 Common method bias test and discriminant validity test

Because all variables were measured using self-report questionnaires, common method bias (CMB) could not be completely ruled out. To reduce potential method bias, participants were assured of anonymity and confidentiality before completing the survey. Following the recommendations of Podsakoff et al. (2003), an unmeasured latent method construct (ULMC) approach was employed to assess the potential influence of CMB.

First, the theoretical measurement model demonstrated a satisfactory fit to the data (*χ*^2^/d*f* = 2.958, CFI = 0.962, TLI = 0.954, RMSEA = 0.064, SRMR = 0.044). Subsequently, a latent common method factor was added and allowed to load on all observed indicators. The method-factor model also demonstrated acceptable fit (*χ*^2^/d*f* = 2.234, CFI = 0.979, TLI = 0.971, RMSEA = 0.051, SRMR = 0.040). The changes in fit indices between the two models were relatively small (∆CFI = 0.017, ∆TLI = 0.017, ∆RMSEA = 0.013, ∆SRMR = 0.004) and remained below the commonly recommended thresholds (CFI/TLI < 0.05; RMSEA/SRMR < 0.015). Therefore, common method bias was unlikely to have substantially affected the findings of the present study.

The AVE values of the five core constructs ranged from 0.538 to 0.798, and all CR values exceeded 0.75, indicating satisfactory convergent validity. To assess discriminant validity, the Heterotrait–Monotrait (HTMT) ratio of correlations was employed. As shown in Table 1, all HTMT values were below the conservative threshold of 0.85 (Henseler et al., 2015), except for the relationship between learning motivation and learning engagement (HTMT = 0.929). To further examine the discriminant validity between learning motivation and learning engagement, an additional chi-square difference test was conducted following the procedure recommended by Cheung et al. (2024). Specifically, the original measurement model was compared with a competing model in which all indicators of learning motivation and learning engagement were specified to load on a single latent construct. The competing model demonstrated a significantly poorer fit than the original measurement model (Δ*χ*^2^ = 52.63, Δd*f* = 4, *p* < 0.001), indicating that learning motivation and learning engagement are empirically distinguishable despite their high correlation. These results provide additional evidence supporting the discriminant validity of the two constructs.

**Table 1:** HTMT results for discriminant validity.

	Hope	GAA	LM	SL	LE
Hope					
GAA	0.447				
LM	0.698	0.814			
SL	0.652	0.522	0.775		
LE	0.721	0.586	0.929	0.739	

GAA, generative AI acceptance; LM, learning motivation; SL, self-directed learning; LE, learning engagement.

Although the HTMT value between learning motivation and learning engagement was relatively high, this finding is theoretically understandable. Learning motivation reflects learners’ reasons, willingness, and intention to engage in learning, whereas learning engagement represents their behavioral, cognitive, and emotional investment in learning activities. According to Self-Determination Theory and previous educational psychology research (Deci and Ryan, 2000), learning motivation is widely regarded as an important antecedent of learning engagement (Chen et al., 2023; Hua, 2019; Wang and Wu, 2025). Therefore, although these constructs are expected to be closely related, they represent conceptually distinct aspects of the learning process. The significant chi-square difference test further supports their empirical distinctiveness.

## 4 Research results

### 4.1 Descriptive statistics and correlations

The results of the Pearson correlation analysis are presented in Table 2. GAA showed a significant positive correlation with LM (*r* = 0.592, *p* < 0.01), SL (*r* = 0.425, *p* < 0.01), hope (*r* = 0.372, *p* < 0.01), and LE (*r* = 0.482, *p* < 0.01). LM was positively correlated with SL (*r* = 0.627, *p* < 0.01), hope (*r* = 0.573, *p* < 0.01), and LE (*r* = 0.748, *p* < 0.01). SL was positively correlated with hope (*r* = 0.598, *p* < 0.01), and LE (*r* = 0.668, *p* < 0.01). Additionally, hope was positively correlated with LE (*r* = 0.671, *p* < 0.01).

**Table 2:** Results of correlation and descriptive statistics analysis.

Variables	GAA	LM	SL	Hope	LE
GAA	1				
LM	0.592^**^	1			
SL	0.425^**^	0.627^**^	1		
Hope	0.372^**^	0.573^**^	0.598^**^	1	
LE	0.482^**^	0.748^**^	0.668^**^	0.671^**^	1
M	3.609	3.621	3.693	3.580	3.737
SD	0.537	0.564	0.730	0.675	0.634
Skewness	0.539	0.341	−0.172	0.346	0.04
Kurtosis	0.409	0.237	0.533	−0.075	0.381

^**^p < 0.01; GAA, generative AI acceptance; LM, learning motivation; SL, self-directed learning; LE, learning engagement.

To assess the potential for multicollinearity, with LE as the dependent variable and GAA, LM, SL, and hope as independent variables, the Variance Inflation Factor (VIF) was calculated for all predictor variables. The results showed that the VIF values ranged from 1.552 to 2.266, which were well below the conservative threshold of 5. Therefore, multicollinearity was not a significant concern in this study.

As a result of the descriptive statistical analysis, the mean values of all five major variables exceeded the median value of 3 points, with learning engagement showing the highest mean among them. Furthermore, the skewness and kurtosis of all variables were within acceptable ranges, indicating that the sample data of this study followed a normal distribution.

### 4.2 Moderated mediation effect

**Figure 2:**
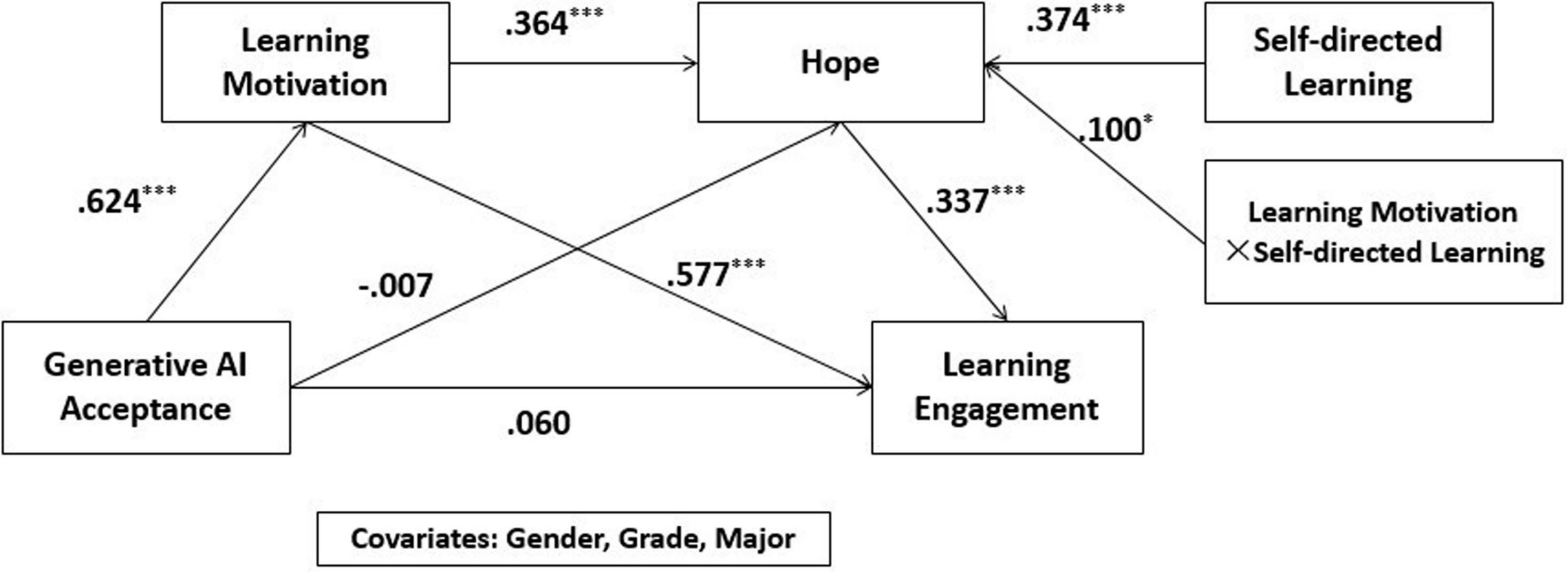
Statistical model of moderated mediation effect.

**Figure 3:**
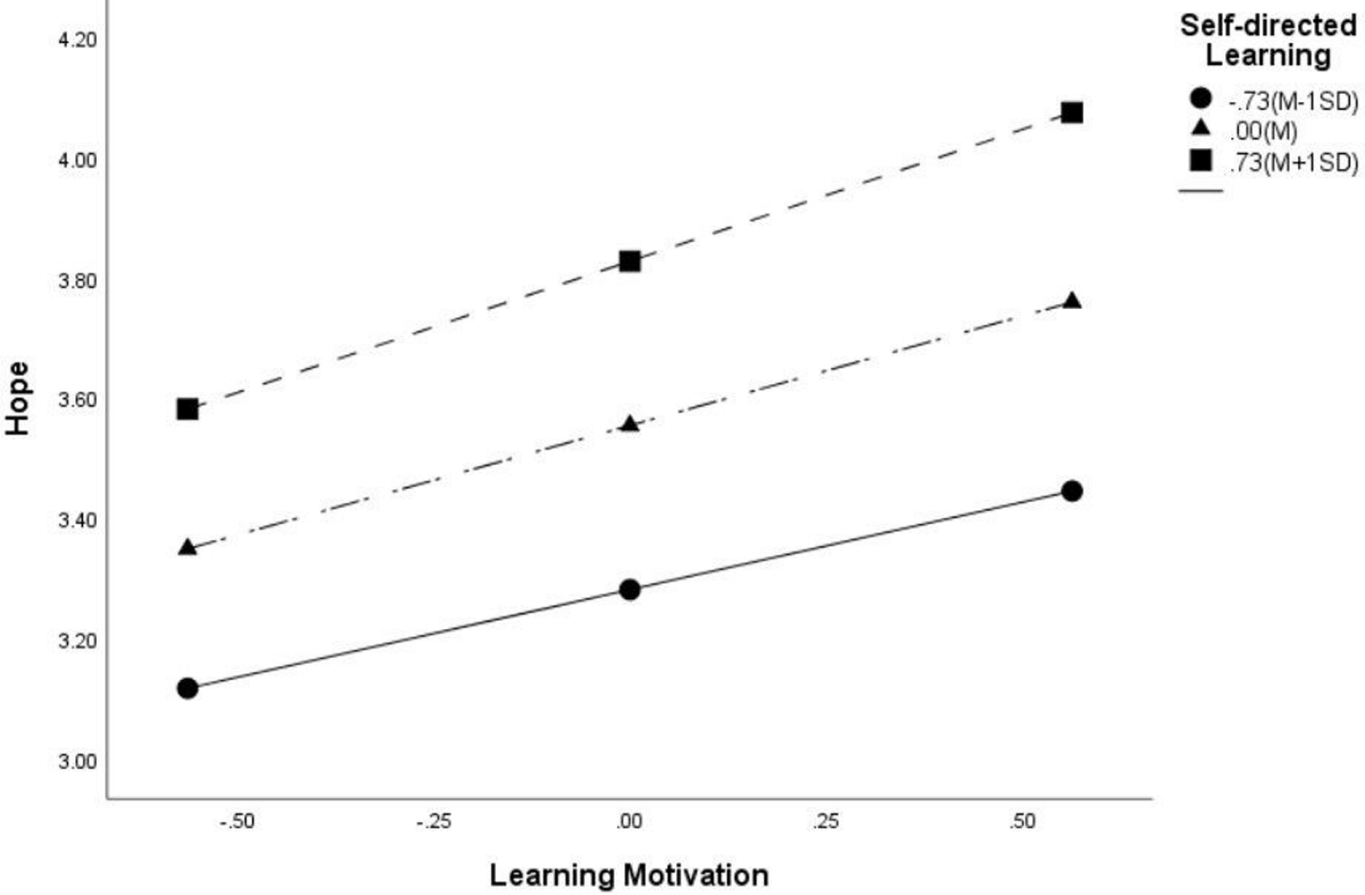
Moderating effect of self-directed learning.

For the moderated mediating effect analysis, the analysis was performed using Model 91 of SPSS PROCESS macro 4.2 proposed by Hayes (2017). The analysis results are presented in Figures 2, 3, Tables 3, 4.

**Table 3:** Results of moderated mediation effect analysis.

Variables		Mediating variable model 1			Mediating variable model 2			Dependent variable model		
		(DV: LM)			(DV: hope)			(DV: LE)		
		Co-effect	SE	*t*-value	Co-effect	SE	*t*-value	Co-effect	SE	*t*-value
Constant		−2.423	0.192	−12.632^***^	3.419	0.250	13.680^***^	2.145	0.209	10.256^***^
ID	GAA	0.624	0.039	15.892^***^	−0.007	0.056	−0.126	0.060	0.040	1.501
M1	LM				0.364	0.060	6.031^***^	0.577	0.043	13.416^***^
M2	Hope							0.337	0.031	10.786^***^
Moderator	SL				0.374	0.042	8.986^***^			
Interaction	LM * SL				0.100	0.048	2.083^*^			
Highest order test	*R*^2^ change				0.005					
	*F*				4.339^*^					
Covariates	Gender	0.046	0.045	1.011	−0.034	0.051	−0.668	0.067	0.038	1.772
	Grade	0.002	0.024	0.071	0.055	0.027	2.023^*^	−0.020	0.020	−0.999
	Major	0.073	0.052	1.404	0.075	0.059	1.265	0.080	0.043	1.854
Model summary	*R* ^2^	0.354			0.434			0.654		
	*F*	64.738^***^			51.562^***^			148.233^***^		

Conditional effects of hope on values of self-directed learning					
SL	Effect (*B*)	SE	*t*-value	LLCI	ULCI
−0.730 (*M* − SD)	0.291	0.073	4.012^***^	0.149	0.434
0.000 (*M*)	0.364	0.060	6.031^***^	0.246	0.483
0.730 (*M* + SD)	0.437	0.067	6.530^***^	0.306	0.569

Moderator values defining Johnson-Neyman significance regions		
SL	% below	% above
−1.620	1.883	98.117

Significant region of the conditional effect of learning motivation according to self-directed learning					
SL	Effect	SE	*t*-value	LLCI	ULCI
−2.693	0.095	0.148	0.641	−0.196	0.385
⁝					
−1.693	0.195	0.106	1.844	−0.013	0.403
−1.620	0.202	0.103	1.965	0.000	0.404
−1.493	0.215	0.098	2.194^*^	0.022	0.407
⁝					
1.307	0.495	0.083	5.965^***^	0.332	0.658

^*^p < 0.05; ^***^p < 0.001; ID, independent variable; DV, dependent variable; *M*, mediating variable; GAA, generative AI acceptance; LM, learning motivation; SL, self-directed learning; LE, learning engagement.

**Table 4:** Analysis of direct and conditional indirect effects.

Direct effect (generative AI acceptance → learning engagement)				
Effect	SE	*t* value	LLCI	ULCI
0.060	0.040	1.501	−0.019	0.139

Conditional and unconditional indirect effects				
Generative AI acceptance → learning motivation → learning engagement				
Effect		Boot SE	Boot LLCI	Boot ULCI
0.360		0.042	0.278	0.442
Generative AI acceptance → hope → learning engagement				
Effect		Boot SE	Boot LLCI	Boot ULCI
−0.002		0.021	−0.044	0.038
Generative AI acceptance → learning motivation → hope → learning engagement				
Self-directed learning	Effect	Boot SE	Boot LLCI	Boot ULCI
−0.730 (*M* − 1SD)	0.061	0.021	0.023	0.104
0.000 (*M*)	0.077	0.019	0.042	0.117
0.730 (*M* + 1SD)	0.092	0.021	0.054	0.138
Index of moderated mediation				
Self-directed learning	Index	Boot SE	Boot LLCI	Boot ULCI
	0.021	0.012	0.001	0.046

In the dependent variable model, generative AI acceptance did not have a significant effect on the learning engagement (*B* = 0.060, *p > 0*.05), but learning motivation had a significant positive effect on learning engagement (*B* = 0.577, *p* < 0.001), and hope had a significant positive effect on learning engagement (*B* = 0.337, *p* < 0.001). The results indicated that the dual mediating effect is significant.

The moderating variable, self-directed learning, has a significant positive effect on hope (*B* = 0.374, *p* < 0.001), and the interaction term between learning motivation and self-directed learning had a significant positive effect on hope (*B* = 0.100, *p* < 0.05). Self-directed learning moderated the effect of learning motivation on hope.

Looking at the conditional effect of learning motivation according to the value of the self-directed learning, which is a moderating variable, on hope, when the self-directed learning is *M* − SD, *B* = 0.291 (0.149 ~ 0.434), when the self-directed learning is *M*, *B* = 0.364 (0.246 ~ 0.483), and when the self-directed learning is *M* + SD, *B* = 0.437 (0.306 ~ 0.569), the conditional effect was significant. These findings suggest that self-directed learning moderated the positive association between learning motivation and hope, with a stronger association observed at higher levels of self-directed learning.

The Johnson-Neiman method was applied to identify the significant region of the conditional effect of learning motivation according to the self-directed learning value, which is a moderating variable. The moderating effect was not significant in the region where the self-directed learning value was lower than −1.620, and 1.883% of the survey subjects fell into this area. In addition, the conditional effect was significant in the area where the self-directed learning was higher than −0.1.620, and 98.117% of the survey subjects fell into this region.

The graph shows the moderating effect of self-directed learning on the effect of learning motivation on hope, as shown in (Figure 3). Under the three conditions of self-directed learning (*M*, *M* ± SD), when learning motivation increased, hope increased, but when the self-directed learning was *M* + SD, the increasing slope was sharp, but when the self-directed learning was *M* − SD, the relatively increasing slope was gentle compared to when it was *M* + SD. That is, even if learning motivation increased equally, hope increased further when the self-directed learning was *M* + SD, but the degree of increase was low when the self-directed learning was *M* − SD.

The direct, conditional, and unconditional indirect effects were analyzed in the path of generative AI acceptance to learning engagement through learning motivation and hope. The direct effect between generative AI acceptance and learning engagement was *B* = 0.060, which was not significant because 0 was included within the 95% confidence interval (−0.019 ~ 0.139). The indirect effect of generative AI acceptance through learning motivation on the learning engagement was *B* = 0.360, which was significant because 0 was not included within the 95% confidence interval (0.278 ~ 0.442). In other words, learning motivation mediates the relationship between generative AI acceptance and learning engagement. The indirect effect of generative AI acceptance on learning engagement through hope was *B* = −0.002; it was not significant because 0 was included within the 95% confidence interval (−0.044 ~ 0.038). Therefore, the indirect association between generative AI acceptance and learning engagement through hope was not supported.

The conditional indirect effect was significant under all three conditions: *B* = 0.092 (0.054 ~ 0.138) when *M* + SD; *B* = 0.077 (0.042 ~ 0.117) when *M*; and *B* = 0.061 (0.023 ~ 0.104) when *M* − SD, with 0 not included in the 95% confidence interval. As a result, the moderated mediating effect of self-directed learning on the path from generative AI acceptance to learning engagement through learning motivation and hope was verified. In addition, the moderated mediating index was 0.021, which was significant because 0 was not included between the upper limit (BootULCI) and the lower limit (BootLLCI) within the 95% confidence interval (0.000 ~ 0.046).

Overall, the findings provided partial support for the proposed hypotheses. Specifically, H1, H2, H4, H5 and H6 were supported, whereas H3 was not supported. The results showed that there is a positive correlation among the five core variables; generative AI acceptance did not directly predict learning engagement, nor did hope independently mediate the relationship between generative AI acceptance and learning engagement. However, learning motivation significantly mediated this relationship, and learning motivation, together with hope, formed a significant sequential mediation pathway linking generative AI acceptance to learning engagement. Furthermore, self-directed learning significantly moderated the relationship between learning motivation and hope, as well as the overall sequential mediation process.

## 5 Discussion

The empirical results reveal significant positive correlations among all five focal variables. Crucially, mediation analysis indicates that the direct effect of generative AI (GenAI) acceptance on learning engagement is statistically non-significant, demonstrating that this relationship is fully mediated. Specifically, the sequential chain mediation pathway—“GenAI acceptance → learning motivation → hope → learning engagement”—is fully validated. Furthermore, self-directed learning significantly moderates the structural path from learning motivation to hope, thereby confirming the hypothesized moderated mediation framework. These findings are contextualized and elaborated below.

### 5.1 Relationships among the core variables

The positive relationship between generative AI acceptance and learning motivation is consistent with the Technology Acceptance Model (TAM), which proposes that individuals who perceive technology as useful and easy to use are more likely to develop favorable attitudes and behavioral intentions toward its use (Davis, 1989). In educational settings, generative AI tools can provide immediate feedback, personalized explanations, and flexible learning support, thereby enhancing learners’ perceived competence and facilitating motivation toward academic tasks (Chiu, 2024). Students who recognize the educational value of AI tools are therefore more likely to invest effort in learning activities.

Furthermore, learning motivation was positively correlated with hope. This finding supports the assumptions of Hope Theory, which posits that motivated individuals are more likely to develop agency thinking and pathway thinking toward goal attainment (Snyder, 2002). When students possess stronger motivation to achieve academic goals, they are more likely to believe that success is attainable and that effective strategies can be identified to overcome learning difficulties. Consequently, motivated learners tend to report higher levels of hope regarding their academic future.

The positive association between hope and learning engagement is also consistent with previous findings indicating that hopeful individuals are more likely to persist when facing challenges, maintain positive expectations, and devote greater cognitive, emotional, and behavioral resources to learning activities (Feldman and Kubota, 2015; Gallagher, 2023). Similarly, students with stronger self-directed learning abilities demonstrated higher levels of motivation, hope, and engagement. This finding aligns with self-directed learning theory, which emphasizes learners’ capacity to regulate their own learning processes through goal setting, strategic planning, self-monitoring, and self-evaluation (Garrison, 1997).

### 5.2 Dual mediating effect of learning motivation and hope

The most important finding of this study is that the association between generative AI acceptance and learning engagement was primarily explained through a sequential motivational–emotional pathway involving learning motivation and hope. Rather than supporting a direct association between technology acceptance and learning engagement, the findings suggest that the educational value of generative AI is associated with learners’ internal psychological processes. Specifically, acceptance of generative AI was associated with higher learning motivation, which in turn was associated with hope, ultimately relating to learning engagement.

This sequential process can be interpreted by integrating the Technology Acceptance Model, Self-Determination Theory, and Hope Theory. According to TAM, technology acceptance reflects learners’ positive evaluations of technological usefulness and ease of use, thereby increasing their willingness to engage with technology-supported learning (Davis, 1989). However, willingness to use technology alone may not be sufficient to explain meaningful educational outcomes. According to Snyder’s (2002) Hope Theory, generative AI acceptance first stimulates learning motivation (providing agency). With this driving force, students are more inclined to use AI to construct problem-solving paths, thereby generating hope, which ultimately translates into learning engagement. This finding supports Self-Determination Theory (SDT), which posits that environmental support (such as effective AI tools) can satisfy students’ competence needs, thereby enhancing motivation (Deci and Ryan, 2000). This transmission mechanism of psychological resources strongly echoes Pan and Zhang (2025), who found that mastery of AI literacy significantly enhances students’ sense of learning efficacy and motivation. The significance of the chain mediation reveals the transmission mechanism of psychological resources. This finding expands on existing literature and highlights that motivation is a necessary prerequisite for hope and behavioral engagement in the context of smart technologies (Luo et al., 2025).

This sequential mechanism also contributes to understanding the educational implications of the Productivity Paradox. The Productivity Paradox suggests that technological investments do not automatically generate improved performance because technological resources must first be transformed into effective organizational or individual capabilities (Brynjolfsson, 1993). Consistent with this perspective, the present findings suggest that acceptance of generative AI alone may not adequately account for differences in learning engagement. Instead, the educational value of generative AI appears to emerge when technological acceptance is accompanied by learners’ motivational and psychological resources. These findings therefore provide a psychological explanation for why previous studies have reported inconsistent associations between generative AI adoption and learning outcomes (Dwivedi et al., 2023; Kasneci et al., 2023).

A particularly noteworthy finding of the present study is that the indirect association between generative AI acceptance and learning engagement through hope alone was not statistically significant, whereas the sequential indirect pathway through learning motivation and hope was significant. This pattern provides important theoretical insight into the psychological processes underlying AI-supported learning and suggests that hope may not function as an independent mechanism linking technology acceptance to learning engagement. According to Hope Theory (Snyder, 2002), hope is a goal-directed cognitive resource composed of two complementary components: agency thinking, referring to the perceived determination to pursue desired goals, and pathways thinking, referring to the perceived ability to generate effective strategies for attaining those goals. Rather than emerging automatically from favorable perceptions of technology, hope develops when learners possess meaningful academic goals and believe they can successfully achieve them through appropriate actions. In contrast, generative AI acceptance primarily reflects learners’ perceptions of the usefulness and ease of using AI technologies in learning (Davis, 1989). Although positive technology acceptance may increase learners’ willingness to use AI-supported learning tools, it does not necessarily strengthen their confidence in achieving academic goals. Consequently, acceptance of generative AI alone may be insufficient to account for variation in hope.

### 5.3 Moderating role of self-directed learning

This study further elucidates that self-directed learning positively moderates the “learning motivation → hope” pathway. Specifically, for students characterized by high SDL capacities, the predictive power of learning motivation on hope is significantly intensified. This finding strongly resonates with Zimmerman’s (2002) self-regulated learning framework. Self-directed learning appears to shape the extent to which motivational resources can develop into positive psychological resources that subsequently become associated with learning engagement. This interpretation complements recent discussions emphasizing that the educational effectiveness of generative AI depends not only on technological capabilities but also on learners’ capacity for self-regulation and reflective learning (Dwivedi et al., 2023; Kasneci et al., 2023). This finding extends previous research by suggesting that self-directed learning functions not merely as an independent predictor of learning engagement but as a boundary condition that facilitates the transformation of motivational resources into goal-directed psychological resources.

In a generative AI environment, learners with high self-directed learning ability are able to plan more clearly how to use AI to meet their learning needs. Therefore, when motivation is aroused, they are more likely to generate the hope of “I can find a way to solve the problem” than students with low self-directed learning (Ingkavara et al., 2022). For example, when students encounter difficulties in understanding a concept, instructors may encourage them to ask AI systems to provide guiding questions rather than direct solutions. Instead of prompting “What is the answer?,” students can be trained to ask, “What concepts should I understand first?” “What alternative approaches can I consider?,” or “What evidence supports this conclusion?.” Such high-agency interactions compel learners to self-monitor their understanding, audit their knowledge deficits, and independently co-construct customized learning pathways, thereby fortifying both self-efficacy and psychological agency. Conversely, students with low self-directed learning, even if motivated, may find it difficult to form a clear sense of a learning path due to a lack of strategies. This means that self-directed learning ability is a key catalyst connecting “wanting to learn” (motivation) and “being confident in learning well” (hope). This finding underscores self-directed learning as a conditional moderation that shapes the effectiveness of generative AI in educational contexts.

### 5.4 Theoretical and practical implications

The present study makes three specific theoretical contributions to the emerging literature on generative AI-supported learning.

First, this study advances current understanding of how generative AI acceptance is associated with learning engagement by identifying a sequential motivational–emotional mechanism involving learning motivation and hope. Previous studies have primarily examined technology acceptance, learning motivation, or hope as independent predictors of educational outcomes. By integrating these constructs within a unified framework, the present findings suggest that motivational and emotional resources operate sequentially rather than independently. This sequential perspective provides a more comprehensive explanation of the psychological processes underlying AI-supported learning.

Second, this study contributes to the ongoing discussion surrounding the Productivity Paradox in educational technology. Previous research has reported inconsistent evidence regarding whether the adoption of advanced technologies necessarily results in improved educational outcomes (Brynjolfsson, 1993). The present findings provide evidence consistent with this perspective by suggesting that acceptance of generative AI alone may not sufficiently account for differences in learning engagement. Instead, the educational value of generative AI appears to depend on learners’ ability to transform technological resources into motivational, psychological, and self-regulatory resources. This perspective contributes to explaining why learners with similar levels of technology acceptance may nevertheless demonstrate different levels of engagement in AI-supported learning environments.

Third, the present study extends self-directed learning theory by identifying self-directed learning as a boundary condition within AI-supported learning rather than merely an individual difference variable associated with academic performance. The findings suggest that self-directed learning strengthens the psychological transformation through which learning motivation becomes associated with hope, highlighting the importance of learner agency in realizing the educational potential of generative AI. This finding enriches current research by emphasizing that effective AI-supported learning depends not only on technology adoption but also on learners’ capacity to regulate and strategically manage their own learning processes.

The present findings also have several practical implications, the results highlight that educators and policymakers should move beyond simply promoting the adoption of generative AI tools. Instead, efforts should focus on cultivating students’ learning motivation, strengthening their sense of hope toward academic goals, and developing self-directed learning competencies. Generative AI should be used not only as a content-generation tool but also as a learning facilitator that supports goal setting, reflective inquiry, and autonomous learning. Such an approach is more likely to maximize the educational benefits of generative AI and foster sustained learning engagement among university students.

## 6 Conclusion

This study advances understanding of the association between generative AI acceptance and learning engagement in higher education by examining the roles of learning motivation, hope, and self-directed learning. The findings indicate that the association between generative AI acceptance and learning engagement was primarily indirect and was statistically explained by a sequential motivational–emotional pathway involving learning motivation and hope. In addition, self-directed learning moderated this pathway, such that the positive association between learning motivation and hope, as well as the sequential indirect association, was stronger at higher levels of self-directed learning. These findings indicate that learners’ motivational, emotional, and self-regulatory resources should be considered together when understanding how generative AI acceptance is associated with meaningful educational outcomes.

Several limitations should be acknowledged. First, the cross-sectional design restricts causal inference regarding the relationships among the variables. Second, the study relied on voluntary participation through an online survey platform and provided a small incentive for questionnaire completion. As a result, self-selection bias cannot be completely ruled out. Students who were more interested in generative AI technologies or more motivated to engage in educational activities may have been more likely to participate in the study. Consequently, the sample may not fully represent the broader population of university students, thereby limiting the external validity and generalizability of the findings. Third, this study was conducted in China; cultural factors such as the education system, technology infrastructure, and social - cultural values might make the results not easily applicable to other cultures. Future research should employ longitudinal, experimental, or mixed-methods designs, utilize multi-source or behavioral indicators of learning engagement, and examine whether the proposed relationships vary across cultural and educational settings. Such efforts would provide a deeper understanding of how generative AI influences learning processes and outcomes in diverse learning environments.

## Data Availability

The original contributions presented in the study are included in the article/supplementary material, further inquiries can be directed to the corresponding author.
